# Cytocompatibility, osteogenic potential, antibacterial and antibiofilm efficacy of a calcium-silicate-based intracanal medication

**DOI:** 10.1007/s10266-025-01143-z

**Published:** 2025-07-04

**Authors:** Sherif Heidar, Shereen N. Raafat, Edgar Schäfer, Shehabeldin Saber

**Affiliations:** 1 Department of Endodontics, Faculty of Dentistry, Suez University, Suez, Egypt; 2 Department of Pharmacology, Faculty of Dentistry, The British University in Egypt (BUE), El Sherouk City, Egypt; 3 Dental Science Research Group, Health Research Centre of Excellence, The British University in Egypt (BUE), El Sherouk City, Egypt; 4 Central Interdisciplinary Ambulance, School of Dentistry, University of Münster, Waldeyerstr. 30, 48149 Münster, Germany; 5 Department of Endodontics, Faculty of Dentistry, The British University in Egypt (BUE), El Sherouk City, Egypt

**Keywords:** Bioceramic, Biofilm, Calcium hydroxide, Confocal laser scanning microscopy, E. faecalis, Intracanal medication

## Abstract

This study assessed the biological and antimicrobial properties of a calcium silicate-based material and calcium hydroxide (CH) when used as intracanal medications. MTT assay, osteogenic differentiation of human periodontal stem cells (hPDLSCs), cell mineralization-assay, and determination of ALP activity were assessed to investigate the biological properties. While the agar well diffusion, crystal violet (CRV) assay and LIVE/DEAD staining of dentin slices infected with a mature *E. faecalis* biofilm were used to assess the antimicrobial properties. Normally distributed data were analyzed using one and two-way ANOVA and post hoc Tukey test, while for non-normally distributed data Kruskal Wallis and Dunn's tests were used. The results showed that both materials were cytocompatible, but BioC-Temp showed statistically higher hPDLSCs viability (*P* < 0.05). hPDLSCs cultured with BioC-Temp extract demonstrated a significantly higher mineralization and more mineralized nodules than CH extract (*P* < 0.05). Both BioC-Temp and CH had similar antibacterial potential against *E. faecalis* in radicular dentin. BioC-Temp has higher mineralization potentials than CH. For the antimicrobial properties, BioC-Temp caused significantly higher inhibition zones than CH (*P* = 0.0001). The biofilm biomass reduction of BioC-Temp was significantly higher than for CH (*P* < 0.05). Regarding the percentage of live *E. faecalis* in biofilm, both BioC-Temp and CH caused significant reductions with no significant difference between them (*P* > 0.05).

## Introduction

Apical periodontitis (AP) is an inflammatory disease caused by a polymicrobial infection of the root canal system [1]. Prevention or treatment of AP is a fundamental objective in endodontics [2]. Effective microbial load reduction during root canal treatment is often complicated by anatomical challenges and microbial virulence factors [3, 4]. Therefore, application of intra canal medications (ICM) between visits is one important clinical strategy implemented to manage AP [5]. ICM should ideally possess potent antimicrobial properties, yet be safe to host tissues [6].

Calcium hydroxide (CH), owing to its favorable biological properties, is the most commonly used ICM [7]. It dissociates into calcium and hydroxyl ions. The former triggers undifferentiated mesenchymal cells essential for tissue healing, while the later establish an antibacterial alkaline microenvironment [8].

Recently, there have been an interest in the use of materials based on calcium silicates [9] in different endodontic applications. Such materials demonstrated excellent biocompatibility [10], bioactivity [11] as well as clinical practicality [11].

Bio-C Temp (Angelus Indústria de Produtos Odontológicos, Londrina, PR, Brazil) is a ready-to-use calcium silicate-based ICM, indicated in cases of conventional root canal treatment and retreatment, apexification and endodontic regeneration [13]. Its composition includes calcium silicates, calcium tungstate, calcium aluminate, calcium oxide, titanium oxide radiopacifiers, and base resin as a vehicle that prevent setting [14]. According to the manufacturer [15], Bio-C Temp does not require frequent replacements providing an advantage over CH.

The biological impacts of Bio-C Temp are not yet fully elucidated. Villa et al. [16] reported that it has a time- and dose-dependent cytotoxicity. Although Benetti et al. [17] reported that it was well tolerated by tissues over time and promoted calcium deposition. Moreover, in vitro studies reported a weaker antimicrobial effect for BioC-Temp in comparison with CH [13]. These findings need confirmation or disproof by more comprehensive investigations.

Therefore, the aim of this study was to compare the cytocompatibility, osteogenic potential antimicrobial and antibiofilm properties of BioC-Temp versus CH. The null hypothesis tested was that there will be no differences between BioC-Temp and CH.

## Materials and methods

Ethics approval for this study protocol was awarded by research ethics committee, British University in Egypt (24–081).

### Biological testing

#### Isolation of hPDLSCs

Impacted mandibular molars were obtained from the Maxillofacial Surgery Department, Faculty of Dentistry, The British University in Egypt. Following extraction, the teeth were preserved in Dulbecco's modified Eagle's medium (DMEM; Sigma, St. Louis, MO, USA) with antibiotics (300 U/ml penicillin and 300 mg/ml streptomycin; Sigma). Isolation of human periodontal ligament stem cells (hPDLSCs) was performed as reported previously [18]. In brief, the periodontal ligaments were fragmented and subjected to enzymatic digestion for 1 h at 37 °C. The digestion process involved the use of culture medium containing collagenase (3 mg/mL) and of dispase II (4 mg/mL). The culture medium consisted of DMEM/F12 (Dulbecco’s Modified Eagle Medium/F12 Ham medium, Sigma), provided with 10% FBS (fetal bovine serum, Gibco BRL, California, USA), and 1% antibiotic/antimycotic (Gibco). Subsequently, the cells were incubated at a temperature of 37 °C, 5% CO_2_ and a humid environment. Cells were observed regularly using an inverted microscope (TCM 400, Labomed, Los Angeles, CA, USA). This study utilized cells at P4 (passage four), with a minimum of three triplicates conducted for each group.

#### Characterization of the Isolated human periodontal ligament stem cells (hPDLSCs)

##### Cell surface markers detection by flow cytometry

Trypsinization was used to detach the hPDLSCs which were analyzed for the presence of specific surface antigens using flow cytometry [19]. In summary, the cells were harvested and treated with 4% paraformaldehyde for a duration of 15 min, cultured with a 3% solution of bovine serum albumin and subsequently exposed to primary antibodies targeting CD45, CD34, CD90, CD73, CD105, and HLA-DR for a duration of 1 h. The cells were rinsed with wash buffer, and the secondary antibodies (BD Biosciences, Piscataway, NJ, USA) were introduced for 45 min at room temperature. Afterwards, the cells underwent three rounds of washing and were subsequently assessed using a flow cytometer (Cytofex, Beckman Coulter Inc., Brea, CA, USA).

### Multilineage differentiation

Multilineage differentiation was performed using a commercially available "Human mesenchymal stem cell functional identification kit" (R&D systems Inc., Minneapolis, MN, USA) as described previously [20]. The kit included specialized media supplements for osteogenesis, adipogenesis, and chondrogenesis. These supplements are designed to stimulate the differentiation of stem cells into osteogenic, adipogenic, and chondrogenic lineages. The cells were cultivated in a 24-well plate using specific media for each kind of differentiation (osteogenic, adipogenic, and chondrogenic) for a duration of 3 weeks. Following the differentiation period, osteogenesis was assessed using Alizarin red staining (Sigma-Aldrich, St. Louis, MO, USA). The process of adipogenesis was assessed using Oil Red O stain solution (Sigma-Aldrich). The chondrogenic differentiation was confirmed using Alcian Blue staining (Sigma-Aldrich).

### Cell viability assessment (MTT assay)

MTT assay was employed to determine the effect of both ICMs on hPDLSCs viability and to determine the optimal concentrations for subsequent experiments. Serial dilutions of the ICMs were prepared, starting with a concentration of 12.5 mg/dl. The prepared concentrations included 12.5 mg/dl, 6.25 mg/dl, and 3.12 mg/dl using the culture medium as diluent. The MTT assay was conducted on days 1, 3 and 7 as previously described [20].

### Osteogenic differentiation of hPDLSCs

Cells were cultured in a 6-well plate using the normal culture medium till reaching 70% confluency. To induce osteogenesis, the osteogenic induction medium was used, which consisted of α-MEM, 10% FBS, dexamethasone 0.1 µM (Sigma), beta-glycerophosphate 10 mM (Merck, Darmstadt, Germany) and ascorbic acid-2-phosphate 2.5 mg/L (Sigma Aldrich, Steinheim, Germany). The study groups were cultured in the osteogenic medium conditioned with the optimal concentration of each material, as determined by the results of the MTT assay [21]. The experiment was conducted over a period of 14 days. The negative control group comprised of cells in normal culture medium, and the positive control group comprised of cells in osteogenic medium without the materials’ extracts. All media were changed twice per week.

### Cell mineralization assay

Alizarin Red S (ARS) staining was used to quantify the degree of mineralization [22]. Following a 14-day period of inducing hPDLSCs for osteogenic differentiation, the osteogenic medium was carefully removed, and the wells were thoroughly washed. The cells were fixed by 10% formaldehyde for 15 min, followed by rinsing the monolayers twice with distilled water. A solution of Alizarine Red stain (pH 4.1) was carefully added to each well, and the plates were incubated at room temperature for 30 min with gentle shaking. The undissolved dye was subsequently removed by aspiration, and the wells were rinsed several times while agitating for 5 min. Following the examination of the mineralized nodules using an inverted microscope, a solution of 10% warm acetic acid (800 mL) was introduced to each well. A 96-well plate was used to transfer aliquots of 150 mL of the supernatant, and the resulting color was measured spectrophotometrically at 405 nm.

### Alkaline phosphatase (ALP) activity

The ALP enzyme's activity was measured by tracking the kinetics of the conversion of colorless para-nitrophenylphosphate (p-NPP) into a vibrant yellow para-nitrophenolate (p-NP) [23]. After inducing osteogenic differentiation in hPDLSCs, the osteogenic media was removed. The monolayers were washed with PBS, followed by a single rinse with alkaline phosphatase buffer (ALPB) at room temperature. One milliliter of ALPB was added to the monolayers on a 6-well plate, followed by the addition of an equal volume of p-NPP solution to each well. Immediately, a volume of 50 μL was extracted and combined with an equal volume of NaOH in a 96-well plate. The preceding stage involved repeating the process every minute for a duration of 10 min for each group. The spectrophotometric measurement was used to determine the yellow color produced by the p-NP at a wavelength of 405 nm. The rate at which p-NP accumulated was graphed over time, and the rate of the reaction was determined by computing the slope of each group.

### Antimicrobial testing

#### Determination of the minimal inhibitory concentration (MIC)

The standard broth serial dilution method [24] was used to determine and compare the MIC of Bio-C Temp and CH (Calcipast, Cerkamed, Poland) against *E. faecalis* ATCC 29212 (American Type Culture Collection). Briefly, plastic microdilution trays that have 96 round bottom wells, were used under strict aseptic conditions and each well contained 0.1 mL Tryptone soy broth (TSB; all media were purchased from Oxoid Ltd, Basingstoke, UK) inoculated with *E. faecalis*, adjusted to a turbidity equivalent to a 0.5 McFarland standard.

From each ICM, 10 mg were dissolved in 1 mL distilled water (1000 µg intracanal medication/mL distilled water). Then, 0.1 mL of each ICM was added to 0.1 mL of TSB suspension in each well. Then serial double fold dilutions of each ICM were done (1.95–1000 µg/mL). For the positive control wells, saline was added instead of ICM. Negative control wells included saline added to TSB without inoculation by *E. faecalis*. The plastic microdilution trays were incubated at 37 °C for 24 h in anaerobic conditions.

Finally, growth of *E. faecalis* was assessed by measuring turbidity. TSB turbidity was measured by a microplate reader (BioTek 800 TS, Santa Clara, United States) at 630 nm (nm). The MIC was recorded as the lowest concentration of ICM that inhibited visible growth of *E. faecalis* in the microdilution tray wells.

#### Determination of minimum bactericidal concentrations (MBC)

Following MIC determination, 25 µL from each ICM dilutions, were inserted on agar plates inoculated with *E. faecalis* and incubated at 37 °C for 48 h in an anaerobic jar using an anaerobic kit. The lowest concentration that inhibited E. faecalis growth on agar plate was recorded as the MBC.

### Antimicrobial activity

The agar well diffusion method [25] was used to evaluate the antimicrobial activity of each ICM in triplicate. Mueller–Hinton agar plates were inoculated by spreading *E. faecalis* on the entire agar surface and incubated for 48 h. Then, a hole with a diameter of 6 mm was punched aseptically with a sterile cork puller, and a volume of 100 µL of each ICM at concentration 10 mg/mL was placed into the well. Agar plates were incubated for 24 h under anaerobic conditions at 37 °C to allow intracanal medication to diffuse into the agar and inhibit the growth of *E. faecalis*. The inhibition zones diameters produced by the different ICM were measured as diameters in millimeters.

### Antibiofilm activity

The crystal violet (CRV) assay [13] was used for antibiofilm assessment of both intracanal medications. An E. faecalis biofilm was grown in a 96-well culture plate, each well contain TSB media, and incubated for 48 h at 37 °C.

Following incubation, each well was washed with 200 µL saline, then 200 µL of each intracanal medicament suspension at sublethal concentrations (25, 50, & 75% of MBC) were added to the wells separately in triplicate, to contact the biofilm. TSB inoculated with *E. faecalis* and medicated with saline only was used as the positive control, and wells with sterile media and no biofilm were used as the negative control.

After 48 h of incubation at 37 °C, the biofilm was stained with 0.1% CRV dye (Labsynth, Diadema, SP, Brazil) for 20 min. Then, excess dye was rinsed with saline, and the plates were dried for 1 h at a temperature of 50 °C to fix the biofilms. Afterwards, 33% acetic acid (200 µL) was added, and the remaining biofilm was quantified according to the optical density using a spectrophotometer at 590 nm (Asys-UVM 340, Biochrom-MikroWin 2000, Cambridge, UK). The results of treatment groups were expressed as percentage reduction in treatment groups as compared to the biofilm mass in the positive control group.

### Confocal laser scanner microscopy (CLSM)

#### Sample size calculation

The sample size was calculated using the results of Wu et al. 2014 [26]. According to this study, the minimally accepted sample size was 16 per group, when mean ± standard deviation of live bacteria (green) in the calcium hydroxide group was 36.63% ± 23.06% (median = 32.55%, range = 4.29%–75.63%) and the estimated mean difference with another group was 25%, when the power was 85% and type I error probability was 0.05. The *t* test was performed by using P.S.Power 3.1.6 (Freeware; Informer Technologies, Los Angeles CA, USA).

### Preparation of the samples

Thirty-two teeth with a single and straight root, caries free and completely formed apices were collected. Teeth were transilluminated under magnification and radiographic imaging was done to discard cracked teeth and roots with multiple canals, then teeth were stored in thymol till use. All teeth were decoronated at the cemento-enamel junction; middle and apical thirds of the root were discarded. Then, the coronal third of each root was splinted longitudinally into two halves, resulting in 64 dentin slices. The cementum of all samples was removed, and root canal walls were polished with 220 to 800 grit silicon carbide papers to create relatively flat surfaces. Using a caliper, dentin slices were checked to have standard dimensions. Thereafter, dentin slices underwent smear layer removal by ultrasonic baths in sodium hypochlorite for 15 min (JK-Dental, Egypt) followed by immersion in distilled water for 10 min, and treatment with 17% ethylenediaminetetraacetic acid (EDTA) (Produits Dentaires SA, Vevey, Switzerland) for 3 min. Finally, samples were sterilized by autoclaving at 121 °C for 20 min.

### Inoculation of extracted teeth with *E. faecalis* and maturation of biofilm

Brain heart infusion (BHI) broth was inoculated with *E. faecalis*, then the broth was adjusted at a turbidity equivalent to 3 × 108 CFU/mL-1 as described by Pereira et al. 2021. Dentin slices were immersed in BHI broth for 3 weeks at 37 °C under anaerobic conditions to develop *E. faecalis* biofilm [27]. During this period, BHI was replenished every 2 days. Negative control dentin slices were immersed in sterile BHI but not inoculated with *E. faecalis*.

### Intracanal medication of samples

Regarding the dentin slices designated for CLSM, the external surfaces were mounted in sterile auto-polymerizing acrylic resin exposing the inner surfaces including the entire root canal lumens for stabilization purpose to medicate root canals. Afterwards, the dentin slices were allocated to 4 groups (n = 16 in each group) according to the intracanal medication: Group A: Bioc-Temp, Group B: CH, Group C: positive control group (infected) and Group D: negative (neither infected nor medicated) control group were treated by plain gel formed by mixing methyl cellulose with distilled water. All slices were incubated anaerobically at 37 °C for 1 week.

Dentin slices were stained by Syto 9/propidium iodide (LIVE/DEAD, BacLight; Invitrogen, Eugene, OR, USA) [28] for assessment of interradicular *E. faecalis* biofilm and antimicrobial potentials of the intracanal medications. Slices were stained with 1:1 mixture of Syto 9 and PI for 15 min, then rinsed by saline, mounted, and directly investigated by CLSM (Leica DMi8, Leica, Mannheim, Germany). The scanning was performed from the top of the biofilm towards dentin surface [29].

Syto 9 is a green, fluorescent stain used for labelling live and dead bacteria. Propidium iodide (PI) is a red fluorescent stain used for labelling only dead bacteria via penetrating damaged membranes and staining the nucleic acid of dead bacteria [28].

### Statistical analysis

Statistical analysis was performed with SPSS 16 ® (Statistical Package for Scientific Studies). Distribution of data was analyzed using Shapiro–Wilk and Kolmogorov–Smirnov test. The data regarding the agar well diffusion and CRV assay were distributed normally. Accordingly, comparison between 2 different groups was performed using the independent *t* test, while comparison between different concentrations was performed by using repeated measures ANOVA followed by Tukey`s post hoc test for multiple comparisons. The effect of total variance assessment in antibiofilm was performed by using two-way ANOVA. Regarding confocal results, the data demonstrated nonparametric distribution, accordingly comparison between groups was performed using Kruskal–Wallis test followed by Dunn's multiple comparisons test. The significance level was set at P ≤ 0.05.

## Results

### Biological testing

#### hPDLSCs isolation and characterization

**Fig. 1:**
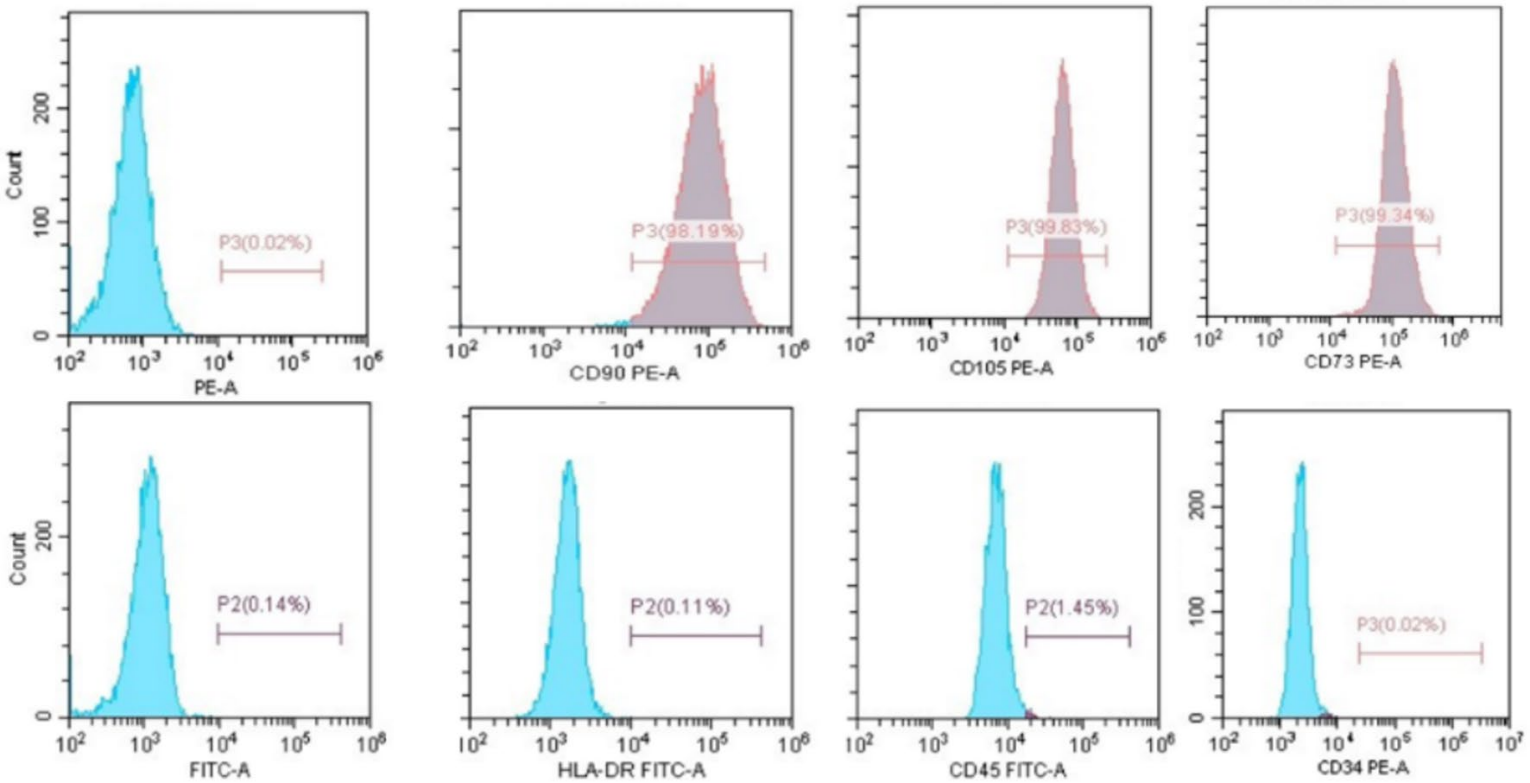
Flowcytometric analysis for the extracted hPDLSCs. Cells population showed 98.19% expression of CD90, 99.83% expression of CD105 and 99.34% expression of CD73, while lacking hematopoietic CDs as HLA-DR, CD45 and CD43 (< 2%)

**Fig. 2:**
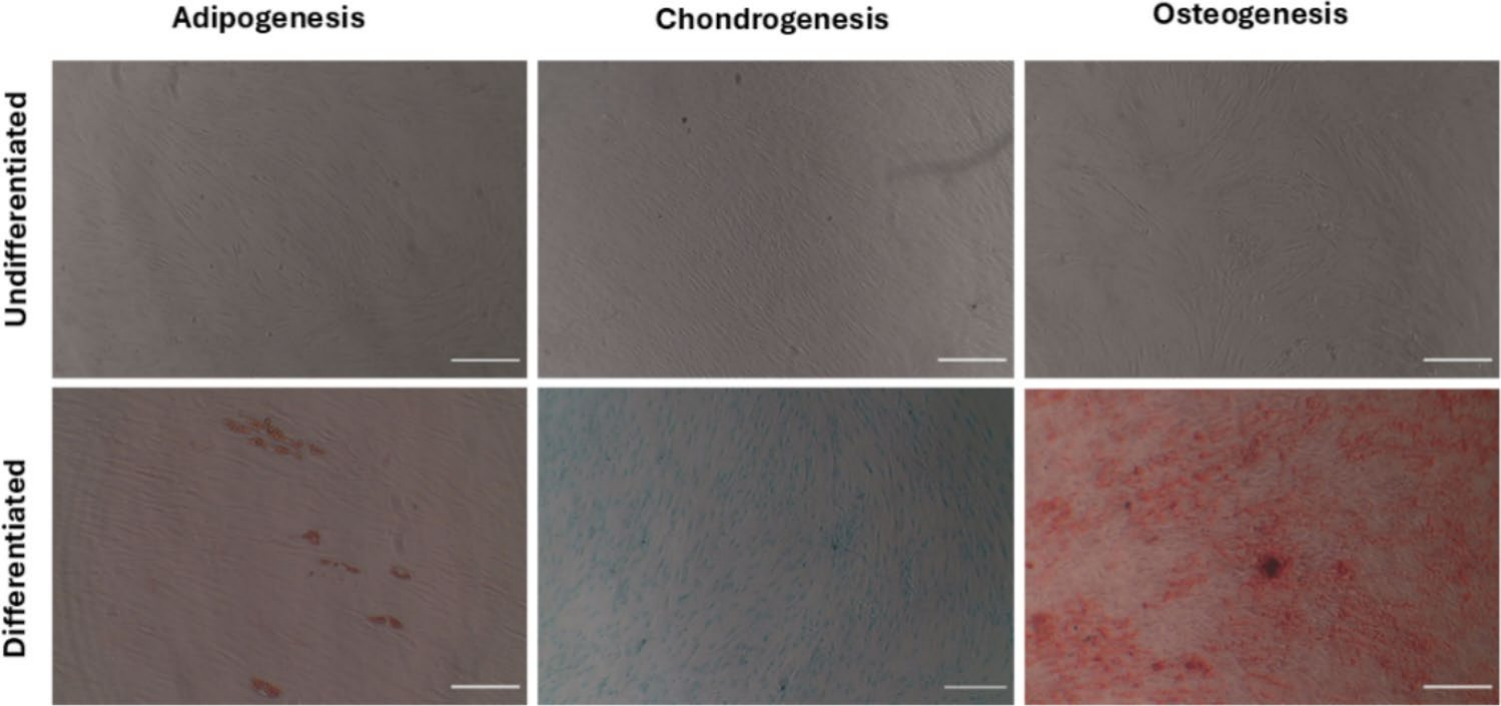
Multilineage differentiation of hPDLSCs into adipocytes (scale bar 125 µm), chondrocytes and osteocytes (scale bar 250 µm)

Flow cytometric analysis of the isolated cell population revealed a positive expression (> 95%) of mesenchymal stem cell markers, including CD105, CD73, and CD90, while showing negative expression (< 2%) of hematopoietic stem cell markers such as HLA-DR, CD43, and CD34 (Fig. 1).The isolated cells demonstrated characteristics of mesenchymal stem cells, exhibiting a spindle shape and adherence to plastic surfaces. These cells successfully differentiated into adipocytes, chondrocytes, and osteoblasts (Fig. 2).

### MTT assay

**Fig. 3:**
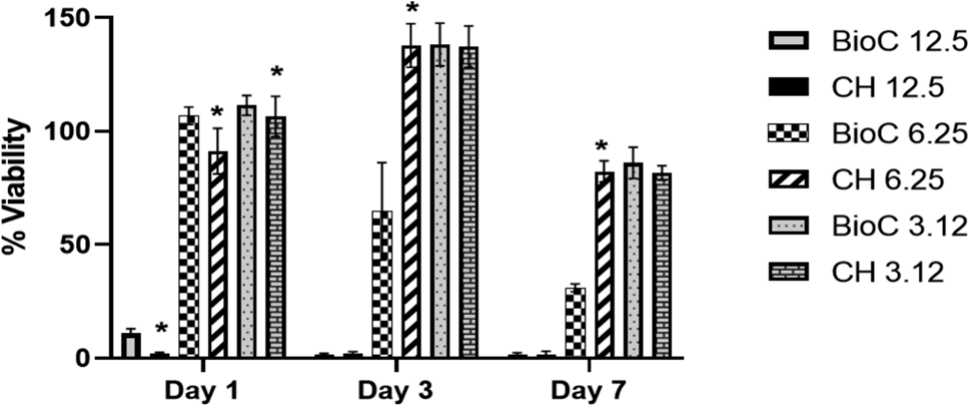
Viability of hPDLSCs following application of each medication at day 1, 3 and 7. * Indicates significant difference between the ICMs at the same time interval

hPDLSCs viability was assessed following incubation with ICM extracts at three concentrations (12.5, 6.25 and 3.12 mg/mL) at three time intervals: Day 1, Day 3 and Day 7 (Fig. 3).

At day 1, the high concentration of both ICMs (12.5 mg/ml) was cytotoxic to the hPDLSCs with no differences between them (p > 0.05). Regarding the other concentrations, both materials were cytocompatible but BioC-Temp showed statistically higher hPDLSCs viability (P < 0.05).

At day 3, the high concentration (12.5 mg/ml) was still cytotoxic as at day 1. At a concentration of 6.25 mg/mL, CH exhibited significantly higher viability (P < 0.05) as compared to BioC-Temp. In contrast, no significant differences were observed in the viability of hPDLSCs at a concentration of 3.12 mg/mL (P > 0.05), with both materials demonstrating high cell viability.

At Day 7, both ICMs remained cytotoxic at the high concentrations. CH demonstrated better cell viability (P < 0.05) at a concentration of 6.25 mg/mL as compared to BioC-Temp, which exhibited viability < 70%. At a concentration of 3.12 mg/mL, no statistically significant difference was observed between the two materials, both showing high cell viability (> 70%). Consequently, the concentration of 3.12 mg/mL was selected to compare the effects of both materials on hPDLSCs osteogenic differentiation.

### ALP activity

**Fig. 4:**
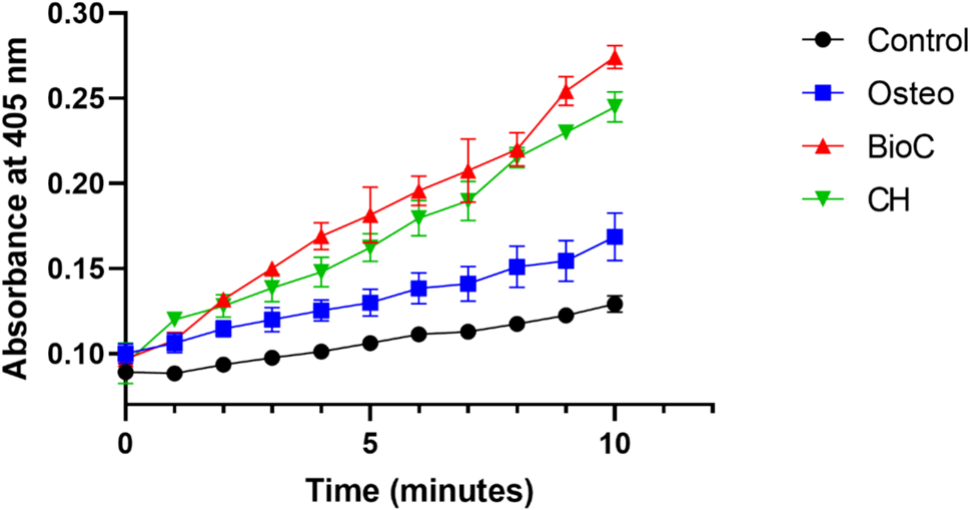
ALP activity of different experimental groups over time

**Fig. 5:**
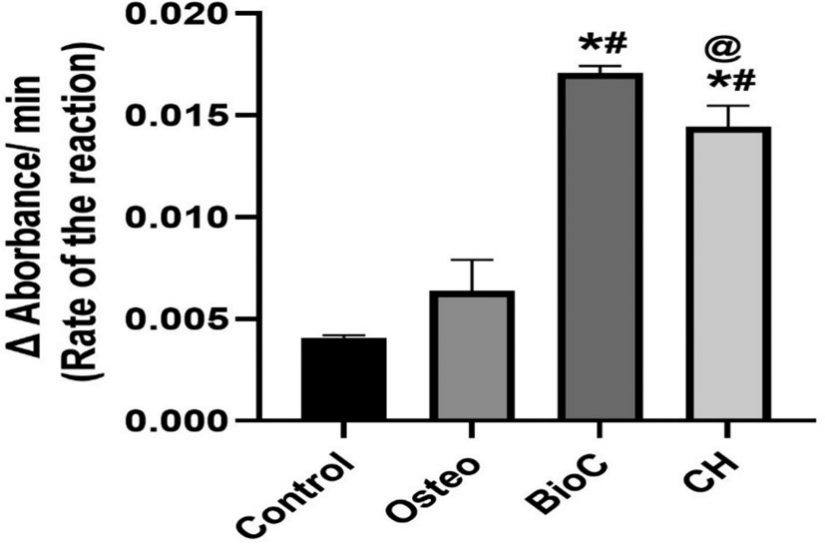
ALP activity among the study groups. * Indicates significant difference compared to the control group, ^#^ Indicates significant difference compared to the Osteo group. ^@^ Indicates significant difference compared to the Bio-C group

hPDLSCs cultured with extracts of both ICMs induced significantly higher mineralization than the Osteo and the negative control groups (P < 0.05) (Figs. 4 and 5). Also, hPDLSCs cultured with BioC-Temp extract demonstrated a significantly higher mineralization potential than those cultured with the CH extract (P < 0.05).

### Cell mineralization assay

**Fig. 6:**
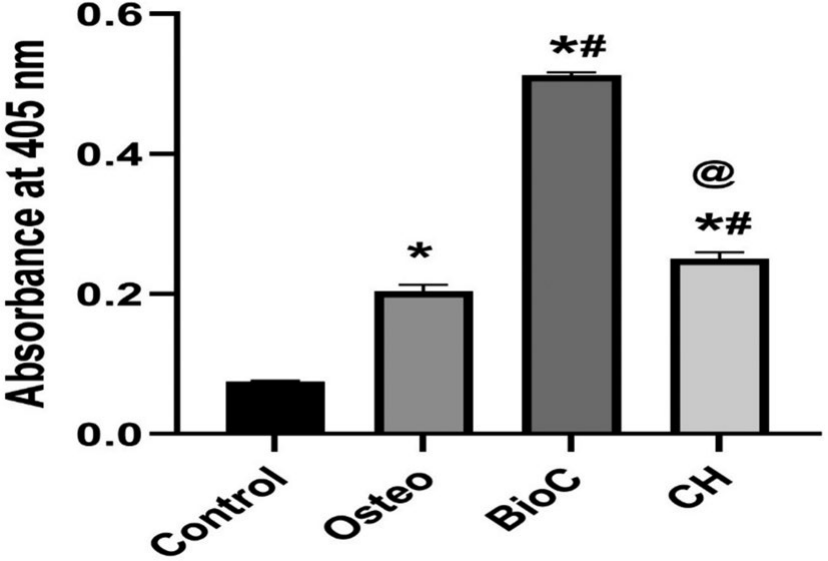
Mineralized nodules detected by ARS staining. Higher mineralized nodules were detected in BioC-Temp. * Indicates significant difference compared to the control group, ^#^ Indicates significant difference compared to the Osteo group. ^@^ Indicates significant difference compared to the Bio-C group

Formation of mineralized nodules through ARS staining (Fig. 6) showed that hPDLSCs cultured with extracts of both ICMs produced significantly more mineralized nodules than the Osteo and the negative control groups (P < 0.05). Moreover, hPDLSCs cultured with BioC-Temp extract demonstrated more mineralized nodules formation than CH (P < 0.05).

### Antimicrobial testing

#### MIC and MBC

The MIC of BioC-Temp and CH against *E. faecalis* were 0.03125 mg/mL and 0.125 mg/mL, respectively. Although the MBC for both ICMs was 0.125 mg/mL.

### Antibacterial activity

Statistical analysis of the results (Table 1) revealed that BioC-Temp showed significantly larger inhibition zones compared to CH (P < 0.0001).

**Table 1:** Comparison between BioC-Temp and CH regarding the inhibition zones in agar wells (expressed in mm)

	Mean	Standard deviation	Difference				
			Mean difference	Std. error difference	95% confidence interval of the difference		P value
					Lower	Upper	
BioC-Temp	28.67	0.58	7.00 mm	0.47 mm	5.69 mm	8.31 mm	0.0001*
CH	21.67	0.58					

^*^Significant effect as P < 0.05

### Antibiofilm activity

Spectrophotometric quantification of CRV dye bound to biofilm was done to assess the mass of remaining biofilms after treatment with ICM at 3 concentrations (25%, 50% & 75% of MBC). The antibiofilm effect of treatment groups was expressed as percentage reduction of biofilm biomass in the treatment groups in comparison with the positive control group.

Inter-group comparison (Table 2) showed that biofilm biomass reduction by BioC-Temp was significantly higher than that by CH at all concentrations (P = 0.001). While the intra-group comparison (Table 3) demonstrated that decreasing concentrations significantly decreased the antibiofilm effect for both ICMs (P = 0.0001).

**Table 2:** Comparison between antibiofilm assessment in the BioC-Temp and CH group at different concentrations regarding biofilm biomass percent reduction (M = mean; SD = standard deviation)

	Concentration (% of MBC)	BioC-Temp group		CH group		Mean Difference	Std. Error Difference	95% Confidence Interval of the Difference		P value
		M	SD	M	SD			Lower	Upper	
Biofilm reduction (%)	25%	82.197	0.590	57.530	0.276	24.67	0.38	23.62	25.71	0.0001*
	50%	90.313	0.445	71.743	0.327	18.57	0.32	17.68	19.46	0.0001*
	75%	96.643	0.627	89.213	0.323	7.43	0.41	6.30	8.56	0.0001*

^*^Significant difference as p < 0.05

**Table 3:** Comparison between anti-biofilm assessment at different concentrations among the same group BioC-Temp group and CH group regarding percentage of biofilm reduction

		25%MBC		50%MBC		75%MBC		P value
		M	SD	M	SD	M	SD	
Biofilm % reduction	BioC-Temp group	82.20^a^	0.59	90.31^b^	0.45	96.64^c^	0.63	0.0001*
	CH group	57.53^a^	0.28	71.74^b^	0.33	89.21^c^	0.32	0.0001*

^*^Significant difference as P < 0.05

Means with different superscript letters per row were significantly different as P < 0.05

Means with different superscript letters per row were significantly different as P < 0.05

A two-way ANOVA analysis (Table 4) was performed to assess the effect of the variants that influence biofilm biomass reduction and showed that changing the effect of drug concentration was more significant than the drug type.

**Table 4:** Two-way ANOVA analysis

Two Way ANOVA test	Source of Variation	% of total variation	P value	Mean square	Sum of squares
Biofilm % reduction	Concentration	51.31	< 0.0001*	798.3	1597
	Medication	41.25	< 0.0001*	1284	1284
	Interaction	7.365	< 0.0001*	114.6	229.2

^*^Significant effect as P < 0.05

### CLSM examination

**Fig. 7:**
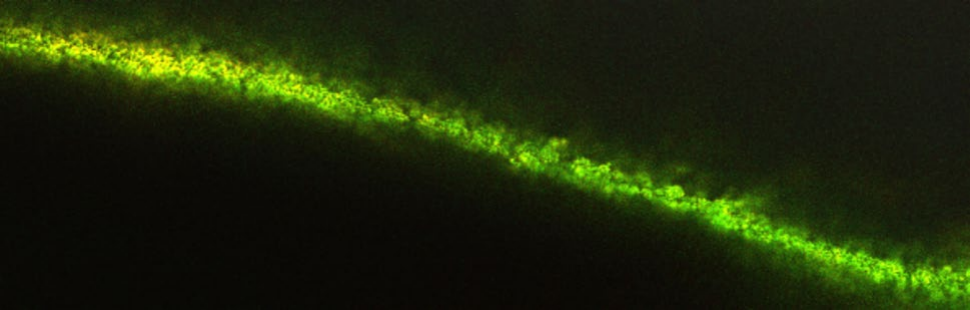
2D CLSM image of *E. faecalis* biofilm in the positive control group, green intensity denoting viable bacteria volume (Magnification 40X)

**Fig. 8:**
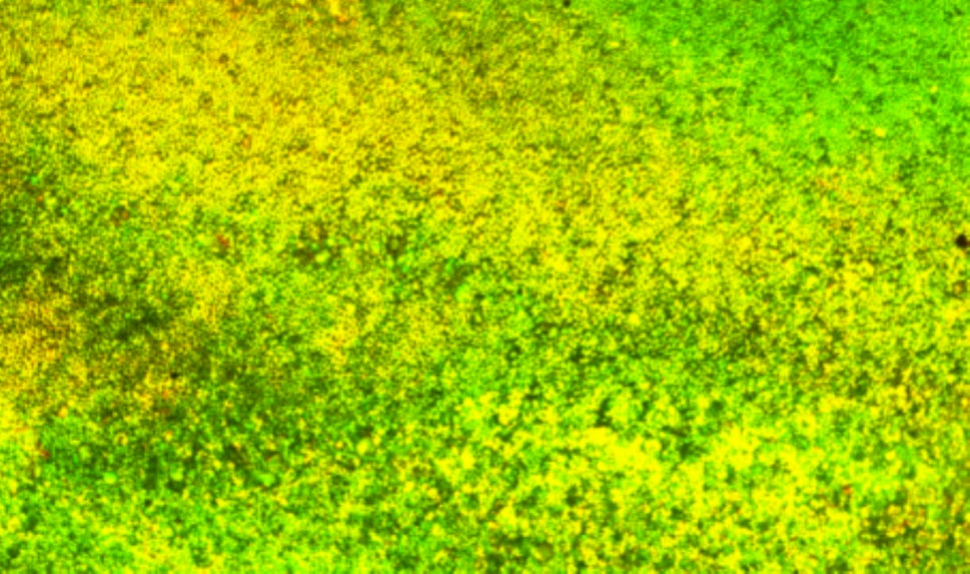
*E. faecalis* biofilm in positive control group with increases green intensity denoting viable bacteria volume. The scanning was performed from the top of the dentin towards the concavity of the root canal lumen (Magnification 40X)

**Fig. 9:**
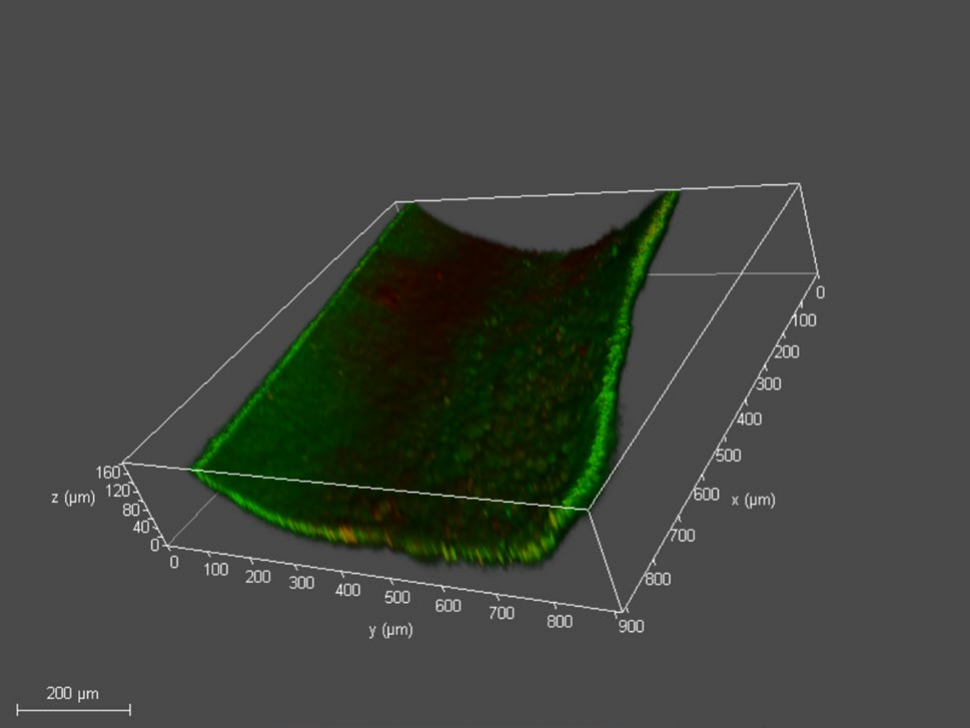
3D image *E. faecalis* biofilm in positive control group captured as mentioned in Fig. 2. The thickness of the *E. faecalis* biofilm in the concave shape attached to the root canal lumen is evident (Magnification 40X)

Two and three dimensional CLSM images were obtained for *E. faecalis* biofilm in the treatment and control groups. The dentin slices of the positive control group showed an *E. faecalis* biofilm with increased green intensity indicating higher values of live bacteria (Figs. 7, 8, 9).

**Fig. 10:**
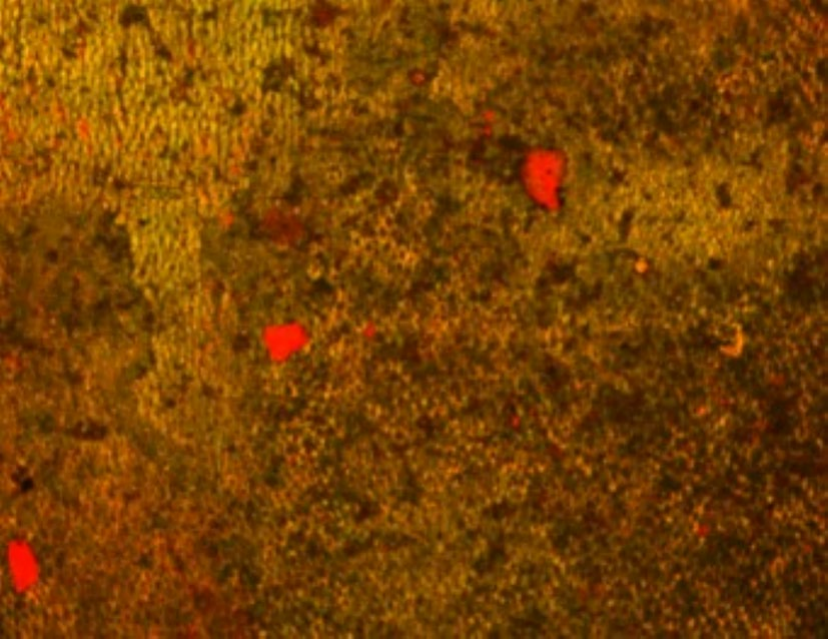
Image of a dentin slice medicated by BioC-Temp. This image was obtained by capturing several images at variable depth due to the concavity of the root canal, then images were integrated by CLSM software to 2D *E. faecalis* biofilm thickness captured by CLSM, red light intensity denoting death of *E. faecalis* (Magnification 40X)

**Fig. 11:**
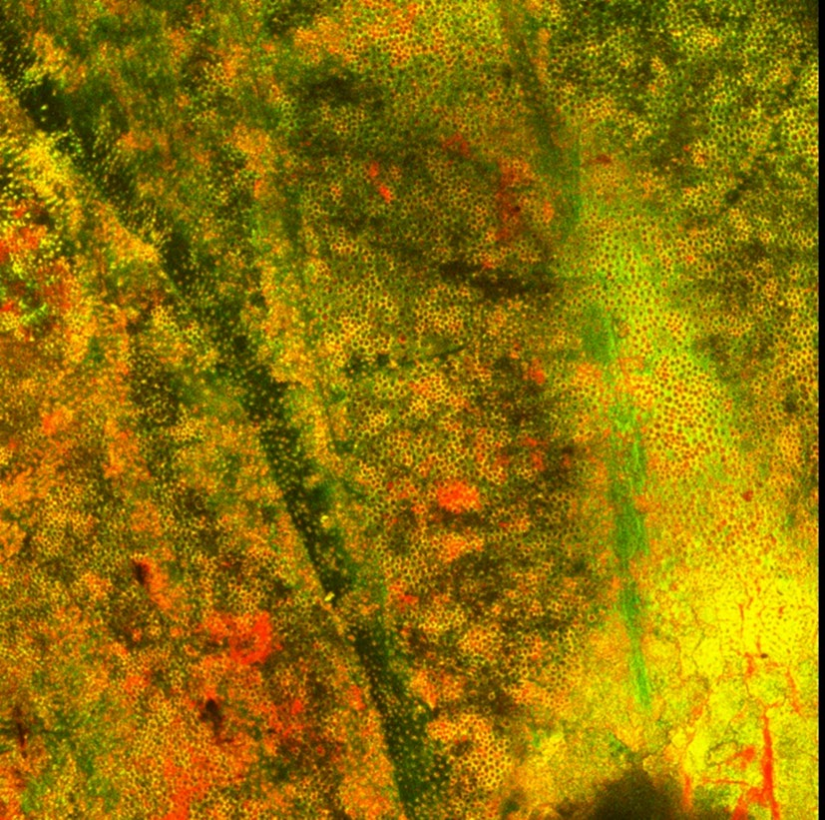
2D image of a dentin slice medicated for 1 week with CH. From the figure increased red color intensity is evident, indicating the increased volume of dead bacteria stained by red PI stain. However, green (live) signifying that still *E. faecalis* can persist even after CH medication (Magnification 40X)

BioC-Temp medicated root canals (Fig. 10) showed an increase in PI red staining intensity as entire portions of the root canal lumen were stained red indicating dead *E. faecalis* bacteria suggesting a biofilm disruption. Although for CH (Fig. 11) mixed red and green zones were evident signifying the presence of some *E. faecalis*.

**Fig. 12:**
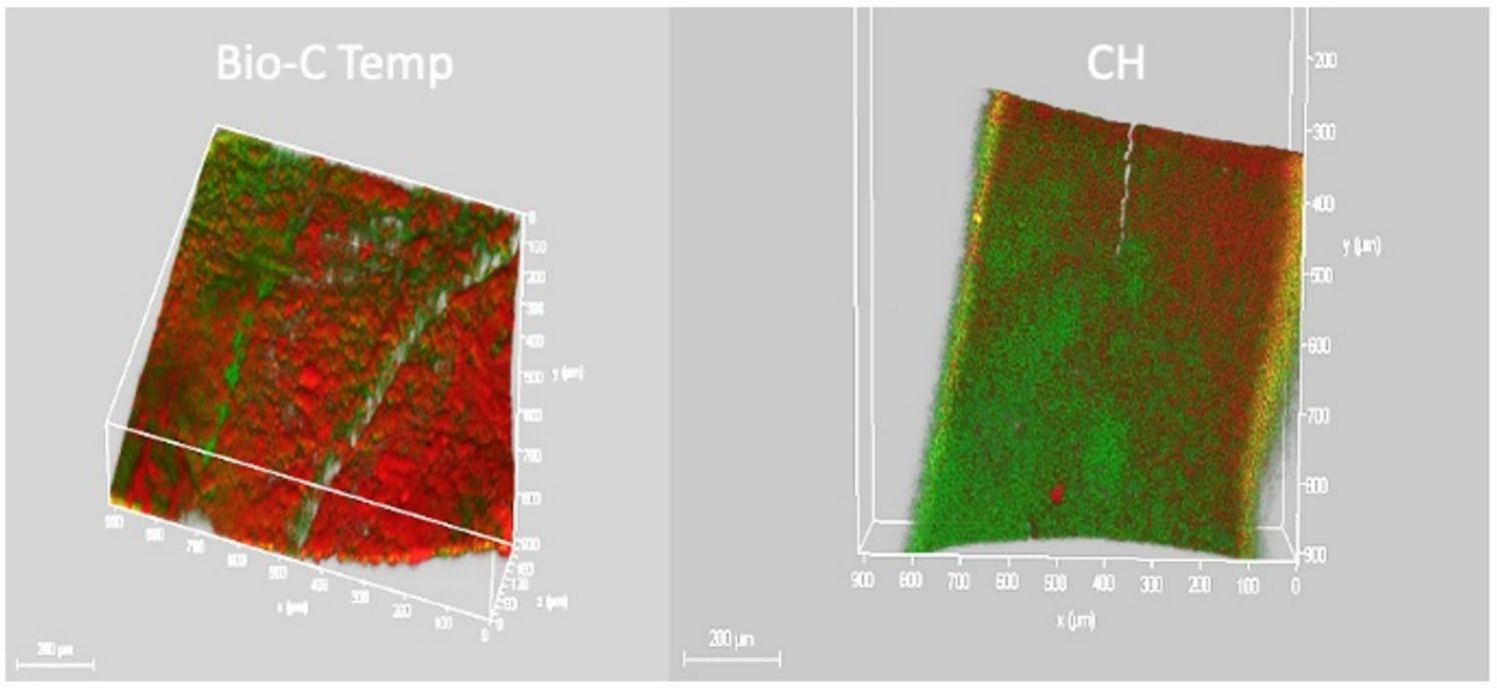
3D image of canal lumens in a dentin slices medicated for 1 week with the ICM. It is evident that BioC-Temp medicated samples displayed more red color signifying a more intense antibiofilm effect

Comparisons demonstrated that both BioC-Temp and CH significantly reduced the percentage of live *E. faecalis* in the dentin slices (47.25% ± 16.86% and 52.95% ± 12.61%, respectively) compared to the control group (93.79% ± 1.27%) (P < 0.0001). However, there was no statistically significant difference between BioC-Temp and CH (P > 0.05) (Table 5 and Fig. 12).

**Table 5:** Comparison between BioC-Temp and CH regarding the percentage of live *E. faecalis* as detected by CLSM (M = mean; SD = standard deviation)

	BioC-Temp		CH		Control		P value
	M	SD	M	SD	M	SD	
Live (%)	47.25^**a**^	16.86	52.95^**a**^	12.61	93.79^**b**^	1.27	0.0001*

^*^Significant difference as P < 0.05

Means with different superscript letters per row were significantly different as P < 0.05

Means with different superscript letters per row were significantly different as P < 0.05

## Discussion

The biofilm model used in this study fulfils the ideal requirements suggested by Esterala et al. [30] for assessment of novel antimicrobial endodontic materials, which are an endodontic pathogen cultured on human dentin as substrate, and an incubation period long enough to allow for biofilm development and maturation.

Regarding the choice of ICM investigated, CH was considered the gold standard as ICM in endodontics and being the most commonly used and investigated. Nevertheless, it does not possess an absolute efficacy against all pathogens [31] and certain levels of microbial resistance have been reported with its use, especially with some resistant microorganisms as *E. faecalis*, which is the biological indicator used in this study [32, 33]. Therefore, the search of new materials with enhanced properties should be always explored. BioC-Temp is declared by its manufacturer solely as an ICM, and therefore this material was used in the present study. Other calcium slicate-based materials (e.g., MTA or Biodentine (Septodont, Saint-Maur-des-fossés, France)) have different physical properties and are intended for other endodontic applications but not declared as an ICM.

Calcium silicate-based materials have stood out for endodontic usage as being biocompatible, noncytotoxic, bioactive and for promoting repair and regeneration [34–36]. Therefore, an ICM based on the same chemistry might be a valid substitute for CH.

The MTT assay evaluated the viability of hPDLSCs cultured with different concentrations of the tested ICM extracts. This was performed to identify the drug concentration that will be both effective and cytocompatible. A pilot study was performed for MTT with starting concentration of 50 mg/mL for both ICMs, the results showed that concentrations of 50 and 25 mg/mL exhibited 0% viability. So, we started the MTT assay in our study with a concentration of 12.5 mg/mL, which was the lowest concentration with high cytotoxicity. The present results showed that CH supported cell viability (above 70% according to ISO standards) at all the observation points in concentrations that were 25% and 50% its MBC, yet 3.12 mg/ml BioC-Temp was significantly the most cytocompatible concentration after 3 and 7 days. As a result, the 3.12 concentration of both ICMs was chosen to compare between their effects on hPDLSCs osteogenic differentiation. This difference in favor of CH can be attributed to the resin base present in BioC-Temp [13]. It has been reported that resin based-calcium silicate materials have inferior biological properties and cytocompatibility [18, 37].

The results of the ALP and ARS assays in the current study demonstrated that BioC-Temp had higher mineralization potentials than CH, Osteo and control. This can be attributed to its high alkalinity, high calcium ions release [16], in addition to the mineralizing potential and documented interfacial activity of calcium silicates when in contact with connective tissues where they can initiate the formation of new healthy bone with osteoblasts at the material–tissue interface [9].

Regarding the in vitro microbiological assays used in this study, the agar diffusion method was used as a primary investigation method. This was augmented by the CRV assay to quantitatively assess the ability of the endodontic pathogen to develop a biofilm. Moreover, CFLSM was used to assess the percentage of viable/dead bacteria after ICM application. The key principle of CFLSM is its ability to differentiate between dead and live bacteria through the permeability of the bacterial cell membrane. Live, viable bacteria have an intact cell membrane that selectively allows the passage of certain molecules in and out of the cell. In contrast, dead or compromised bacterial cells have a disrupted or permeable cell membrane.

The results obtained from the microbiological assays showed that compared to CH, BioC-Temp showed significantly larger inhibition zones (*P* < 0.0001), significantly more biofilm biomass reduction at all concentrations (*P* = 0.001), and a significantly less percentage of live bacteria (*P* < 0.0001).

These effects can be attributed to the material alkalinity [38], its favorable physicochemical interactions with the dentin interface [39], and a higher tubular penetration (depth and area) than CH [40]. Conversely, the present findings disagree with those of Balto et al. 2024 [41], who reported that after application for 1 week, BioC-Temp exhibited a significantly lower percentage of dead bacteria compared to CH, but this was against *Fusobacterium nucleatum*. Also, Guerreiro et al. [13] reported a lesser antibacterial activity for BioC-Temp versus CH, but this was using the direct contact test using planktonic bacteria. These controversial findings amongst studies highlight the need for a standardized multispecies mature biofilm model for testing the antimicrobial efficacy of endodontic materials.

Regarding the intra-group comparison, the present results showed that by decreasing the ICM concentrations, the antibiofilm effect for both ICMs also decreased significantly (*P* = 0.0001). Furthermore, the two-way ANOVA analysis performed to assess the effect of the variants on biofilm biomass reduction revealed that the drug concentration was more significant than the drug type.

Regarding the limitations of this study, using a single resistant pathogen in an ex vivo model may not accurately reflect the complex oral microbiota. It is critical to recognize that the effectiveness of ICMs utilized in endodontic therapy may vary between laboratory and clinical situations. The laboratory environment is strictly regulated, with certain pH, temperature, and nutrient requirements. whereas the in vivo environment is more complex and influenced by a number of variables, such as microbial synergistic partnerships, the host's immunological response, and the buffering capabilities of dentin. As a result, choosing the right concentrations for ICM's clinical application is essential to guaranteeing both the materials' cytocompatibility and the continuation of their biological effects.

## Conclusions

BioC-Temp represents a comparable alternative to CH regarding the antimicrobial effect in dentin against a mature *E. faecalis* biofilm. Moreover, BioC-Temp has higher mineralization potentials than CH.

## Data Availability

Available upon reasonable request from the corresponding author.
